# Molecular characterization of HIV-1 genome in fission yeast *Schizosaccharomyces pombe*

**DOI:** 10.1186/s13578-015-0037-7

**Published:** 2015-08-25

**Authors:** Joseph Nkeze, Lin Li, Zsigmond Benko, Ge Li, Richard Y Zhao

**Affiliations:** Division of Molecular Pathology, Department of Pathology, University of Maryland School of Medicine, Baltimore, MD 21201-1192 USA; Department of Microbiology and Immunology, University of Maryland School of Medicine, Baltimore, MD 21201-1192 USA; Institute of Human Virology, University of Maryland School of Medicine, Baltimore, MD 21201-1192 USA; AIDS Research Department, Beijing Institute of Microbiology and Epidemiology, Beijing, 100071 China; Department of Chromosome Biology, Max F. Perutz Laboratories, University of Vienna, Vienna, Austria

**Keywords:** HIV-1, Viral genome, Gene expression, Functional analysis, Fission yeast, *Schizosaccharomyces pombe*, Subcellular localization, Cellular proliferation, Colony formation, Oxidative stress, Mammalian cell

## Abstract

**Background:**

The human immunodeficiency virus type 1 (HIV-1) genome (~9 kb RNA) is flanked by two long terminal repeats (LTR) promoter regions with nine open reading frames, which encode Gag, Pol and Env polyproteins, four accessory proteins (Vpu, Vif, Vpr, Nef) and two regulatory proteins (Rev, Tat). In this study, we carried out a genome-wide and functional analysis of the HIV-1 genome in fission yeast (*Schizosaccharomyces pombe*).

**Results:**

Each one of the HIV-1 genes was cloned and expressed individually in fission yeast. Subcellular localization of each viral protein was first examined. The effect of protein expression on cellular proliferation and colony formations, an indication of cytotoxicity, were observed. Overall, there is a general correlation of subcellular localization of each viral protein between fission yeast and mammalian cells. Three viral proteins, viral protein R (Vpr), protease (PR) and regulator of expression of viral protein (Rev), were found to inhibit cellular proliferation. Rev was chosen for further analysis in fission yeast and mammalian cells. Consistent with the observation in fission yeast, expression of HIV-1 *rev* gene also caused growth retardation in mammalian cells. However, the observed growth delay was neither due to the cytotoxic effect nor due to alterations in cell cycling. Mechanistic testing of the Rev effect suggests it triggers transient induction of cellular oxidative stress.

**Conclusions:**

Some of the behavioral and functional similarities of Rev between fission yeast and mammalian cells suggest fission yeast might be a useful model system for further studies of molecular functions of Rev and other HIV-1 viral proteins.

## Background

**Fig. 1:**
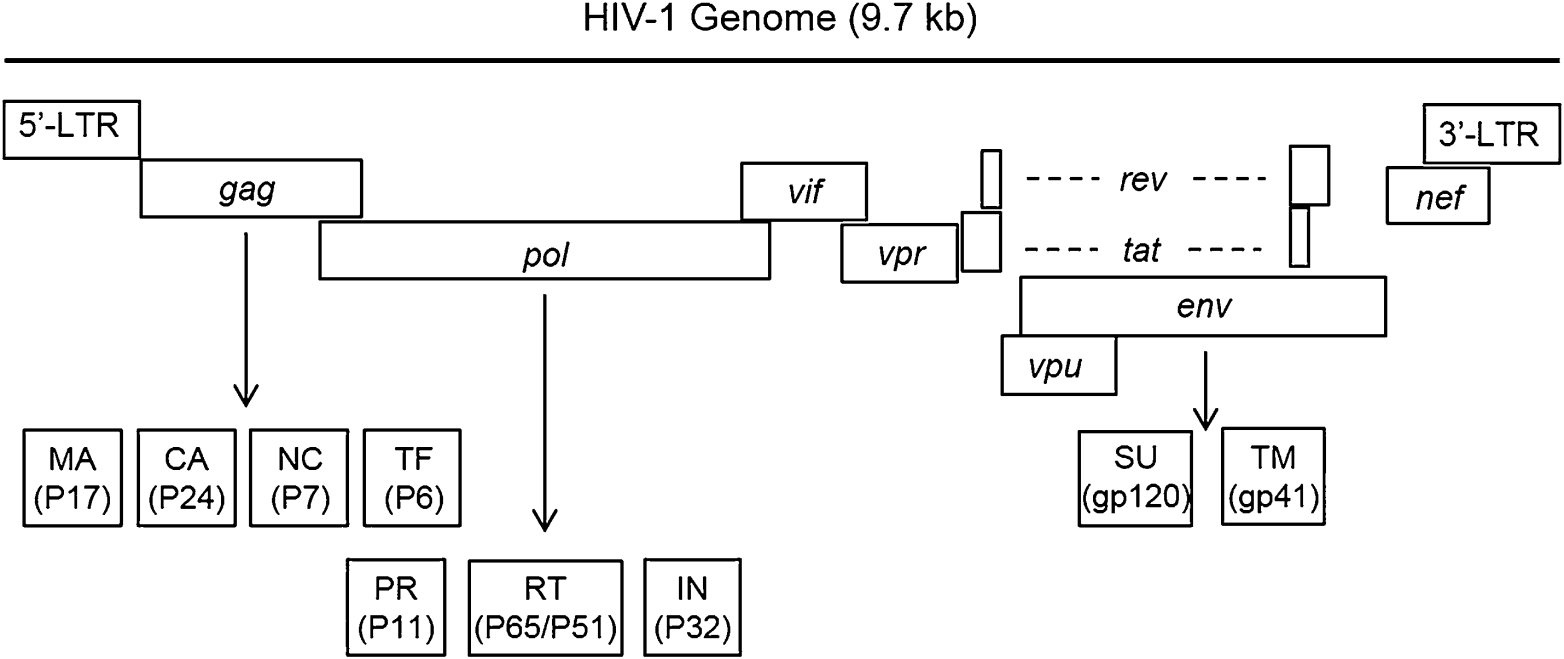
Schematic diagram of HIV-1 genome. The total size of HIV-1 genome is approximately 9.7 kb. Each of the viral genes is drawing based on the relative orientation in the entire RNA genome. *Arrows* points to cleaved protein products. *Dashed lines* represent RNA splicing. The *number in parenthesis* is molecular weight of each protein. *LTR* long-term repeat, *Gag* group-specific antigen, *MA* matrix protein, *CA* capsid domain, *NC* nucleocapsid, *TF* trans-frame protein, *Pol* polymerases, *PR* protease, *RT* reverse transcriptase, *IN* integrase, *Env* envelope protein, *SU* surface membrane protein, *TM* trans-membrane protein, *Vif* viral infectivity factor, *Vpr* viral protein R, *Vpu* viral protein U, *Nef* negative regulatory factor, *Rev* regulator of expression of viral proteins, *Tat* trans-activator of transcription.

The human immunodeficiency virus type 1 (HIV-1), like other retroviruses, is made up of an RNA encoded genome of approximately 9.7 kilobases (kb). Both ends of the RNA genome are flanked by a long terminal repeat (LTR) promoter region (Fig. 1). Between the two LTR regions, there are three polyproteins (Gag, Pol, Env), four accessory proteins (Vpu, Vif, Vpr, Nef) and two regulatory proteins (Rev, Tat). HIV-1 RNA also contains regulatory regions which are important for transcription initiation and polyadenylation.

The polyprotein Gag is required for both virion assembly and maturation [1, 2]. The Gag protein is cleaved by viral protease into P17, P24, P7, and P6 proteins shortly after budding from the host cell [1]. P17 (MA) protein lies in the inner surface membrane of matured viral particles [3]. It is important for RNA targeting of the plasma membrane prior to viral assembly, incorporation of the Env glycoprotein into the viral particle [4, 5] and the particle release [6, 7]. P24 (Capsid domain, CA) protein forms a shell surrounding the viral RNA genome and core-associated proteins in mature virion. It plays various roles including incorporation of the Gag-Pol precursor into virions during viral assembly [8], recruitment of the viral infectivity enhancing protein Cyclophilin A (CypA) [9–11], and early post entry [12]. P7 (Nucleocapsid, NC) plays important roles in the encapsulation and protection of viral RNA, promotion of viral assembly and in early post entry steps including reverse transcription [13]. P6 is important for Vpr packaging into the viral particle and virus budding from the cell membrane [14].

The Pol protein is expressed as a Gag–Pol fusion product since its gene lacks an initiation codon. It is subsequently cleaved by HIV-1 protease to produce MA, CA, NC, trans-frame protein (TF), viral enzymes protease (PR), reverse transcriptase (RT), and integrase (IN) [15]. PR cleaves the Gag and Pol precursors thus rendering the virion infectious [16]. RT is an asymmetric heterodimer with its main role to reverse transcribe viral RNA into pro-viral DNA prior to viral integration to host chromosomes [17]. Other functions of RT include RNA-directed DNA polymerase, DNA directed DNA polymerase and ribonuclease hybrid activities (RNase H) [18]. IN is active only as a tetramer and it is responsible for the integration of the linear double-stranded proviral DNA into the host cell chromosome [19].

The Env/gp160 protein is a precursor protein encoded by a spliced mRNA, which is subsequently cleaved by cellular proteases into the envelope gp120 surface membrane protein (SU) and gp41 trans-membrane protein (TM) [20]. The gp120 surface subunit harbors the N-terminal of the Gp160. It binds to cell receptors attaching virus to target cells and also regulates viral entry. Gp41 is the C-terminal 345-amino acid protein of gp160. It is also involved in the viral entry and mediation of fusion [21].

In addition to the retroviral Gag, Pol, and Env proteins, HIV-l produces four accessory proteins, i.e. Nef, Vif, Vpr and Vpu, and two regulatory protein, i.e. Tat, and Rev [22]. While Tat and Rev are required for viral replication, Nef, Vif, Vpr and Vpu are dispensable for viral proliferation in many of the in vitro systems [23, 24]. However, they are often necessary for viral replication and pathogenesis in vivo and for many of the essential viral functions during the viral life cycle.

The fission yeast *Schizosaccharomyces pombe* (*S. pombe*) is a unicellular eukaryote of the Division Ascomycota. It is cylindrical and rounded at both ends. It reproduces meiotically by ascospores and proliferates asexually by cell division (fission). Its length and diameter are about 7–12 µm and 3–4 µm, respectively [25, 26]. It has a relative small genomic size of about 1.5 × 1 0^7^ bp in the haploid state [27]. *S.pombe* has many of the same fundamental cellular features as larger multicellular organisms which makes it very useful in many of the molecular-biological studies [25–28]. It contains gene slicing mechanism that is able to remove introns from genes of higher eukaryote and mammals [25, 27, 29]. Its signal-transduction system is able to transmit signals from the mating factor receptor through a G-protein-coupled system to the effectors [25]. The cell cycle is also similar to that in higher eukaryotes [27, 31]. For more than three decades, fission yeast has been used in many studies to investigate the structures, functions and expression of eukaryotic genes, especially from mammalian origins [25, 27, 29, 31]. It should also be mentioned that our laboratory has been using fission yeast as a model system to study the effect of HIV-1 Vpr on basic cellular functions in the past 20 years, which includes cell cycle G2/M regulation, nuclear transport and induction of cell death and apoptosis [31–36]. Results of those earlier studies have demonstrated the effects of Vpr are resembled, most of the time, to those of found in mammalian cells. Furthermore, this simple model system allowed us to discover new Vpr-specific activities that are otherwise difficult to uncover solely based on mammalian studies [37–41]. Our intention in this study was therefore to expand and to test whether we could also use fission yeast as a host system to study other HIV-1 proteins.

It should be mentioned that both fission yeast and budding yeast (*Saccharomyces cerevisiae*) have long been used to study various HIV-1 proteins. Some specific examples include HIV-1 Gag [31], Tat [32], Rev [33], Vpr [34], Vpx [35], Vpu [36], Integrase [42] and Protease [43], respectively. A number of reviews have also described the use of yeast as a model system for HIV-1 studies [44–47]. However, none of those early yeast studies has studied the whole HIV-1 genome all at once. Therefore our goal here was to carry out a genome-wide and functional analysis by cloning and expressing every viral gene encoded by the HIV genome in fission yeast. Subcellular localizations and effects of these viral proteins on fission yeast cell proliferation and colony formation, an indication of cytotoxicity, were observed and analyzed.

## Results

### Subcellular localizations of HIV-1 proteins in fission yeast

**Fig. 2:**
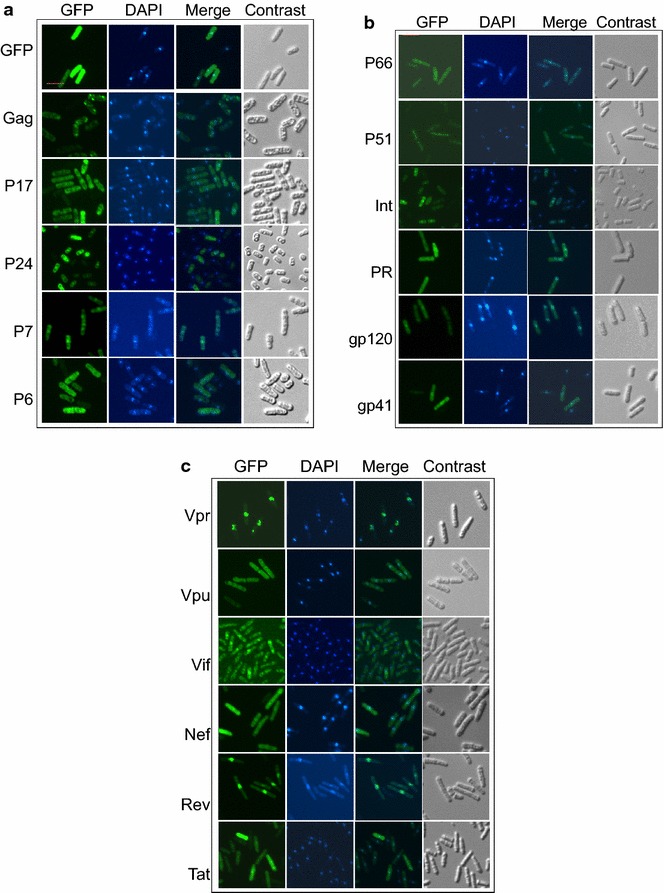
Subcellular localization of HIV-1 proteins in fission yeast. Fission yeast strains expressing normal GFP or N-terminally GFP-tagged HIV-1 proteins were grown to a log phase in EMM selective media. The cells were re-inoculated into fresh media without thiamine (to induce gene expression) and grown for 24–30 h. The nuclei were stained with DAPI. The cells were examined using fluorescence microscopy for subcellular localizations of the GFP-tagged proteins with stained cellular nuclei. *Each column* represents different microscopic views: GFP, for protein subcellular location; DAPI, for localization of the nucleus; Merge, merging images of GFP and DAPI; and the “Contrast” is for the overall view of the cell. The Gag gene products are shown in **a**, the Pol and Env gene products in **b**, and the auxiliary and regulatory proteins in **c**. The *scale bar* represents 10 μm.

In order to determine the subcellular localization of HIV-1 proteins in fission yeast, SP223 cells were transformed with a fission yeast expression pYZ3N plasmid producing each of the HIV-1 viral protein sequences in fusion with an N-terminal GFP [48]. The fission yeast strains containing different viral proteins expressing plasmids were inoculated into liquid selective medium and the protein expression was induced following the removal of thiamine from the growth medium as described previously [34, 49]. After cultured for 24–30 h (depending on the intensity of green fluorescence), the GFP-viral fusion protein products were observed under a fluorescence microscope. As shown in Fig. 2a, GFP alone disperses throughout the cells indicating no preference of subcellular location. In contrast, the Gag protein aggregated in the cytoplasm of unknown sites. P17 protein was excluded from the nucleus and localized exclusively in the cytoplasm. P24 protein was localized both in the nucleus and cell membrane, while the P7 protein was predominantly localized in the nucleus. P6 was found distributed throughout the cell. The localizations of Pol proteins (P66, P51, IN, PR) and Env proteins (including Gp120 and Gp41) were monitored and shown in Fig. 2b. P66 was found more in the nucleus than in the cytoplasm; whereas P51 proteins were found exclusively in the cytoplasm. Consistent with the role of HIV-1 integrase, IN was indeed found more in the nucleus than in the cytoplasm. PR was distributed evenly throughout the cell, while Gp120 and Gp41 were localized more in the nucleus than in the cytoplasm. The subcellular distribution of HIV-1 accessory proteins (Vpr, Vpu, Vif, Nef, Rev, and Tat) was shown in Fig. 2c. Consistent with previous findings with Vpr [48], it localizes predominantly in the nucleus. Similarly, Vif, Rev, and Tat all accumulated in the nucleus of yeast cells. Nuclear localization of Tat has also been reported in budding yeast previously [50]. Vpu in cytoplasm but Nef was distributed throughout the cell. Finally, the subcellular location patterns observed in fission yeast were compared with that reported from mammalian cell studies. As summarized in Table 1, overall, there is a general correlation of the subcellular localization of each viral protein between fission yeast and that of previously reported in mammalian cells.

**Table 1:** Subcellular localization of HIV-1 proteins in mammalian and fission yeast cells

HIV-1 protein	Subcellular localization	
	Mammalian (references)	Fission yeast (this study)
Gag	Cytoplasmic membrane [51]	Cytoplasm
P17	Cytoplasm, membrane [52]	Cytoplasm
P24	Cytoplasmic membrane [53]	Nucleus and cell membrane
P7	Nucleus [54]	Nucleus
P6	Cytoplasmic membrane [55]	Throughout cell^a^
P66	Cytoplasm [56]	Nuclear > cytoplasm
P51	Cytoplasm [56]	Cytoplasm
Integrase	Nucleus [57]	Nuclear > cytoplasm
Protease	No report found	Throughout cell
Gp120	No report found	Nuclear > cytoplasm
Gp41	No report found	Nuclear > cytoplasm
Vpr	Nucleic membrane [48]	Nucleus [58]
Vpu	Cytoplasm [59]	Cytoplasm
Vif	Nucleus [60]	Nucleus
Nef	Cytoplasm [61]	Throughout cell^a^
Rev	Nucleus [62]	Nucleus
Tat	Nucleus [63]	Nucleus

^a^Different subcellular localization pattern was observed.

### Effects of HIV-1 gene expression on yeast colony formation

**Fig. 3:**
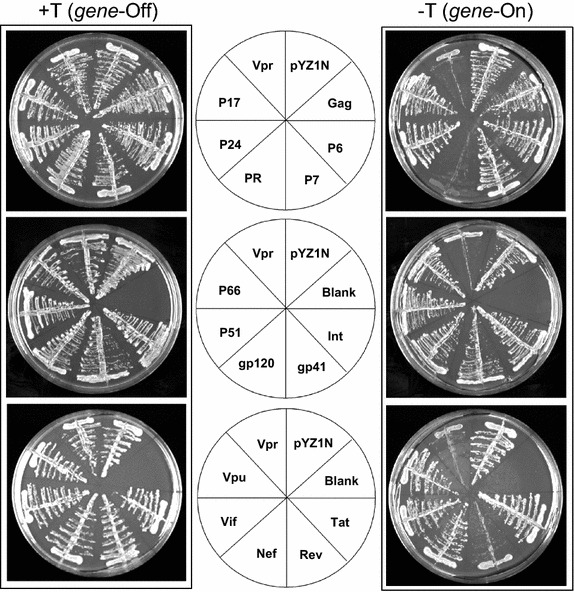
Effects of HIV-1 viral proteins on colony formation. Wild type *S. pombe* cells containing HIV-1 viral gene-expressing plasmids were grown overnight in EMM liquid medium supplemented with thiamine to mid-log phase. The following day, cells were recovered, re-suspended in fresh media and streaked onto the minimal selection EMM agar plates with thiamine (*left* +T plates; no gene expression) or without thiamine (*right* −T plates; with gene expression). The plates were incubated at 30°C for 6 days before observation. The pYZ1N-*vpr* and pYZ1N plasmids were used as positive and negative controls, respectively [34].

To determine potential cytotoxic effect of HIV-1 viral protein expression on fission yeast, yeast colony formation, an indication of cellular growth and potential cytotoxicity, was measured. Fission yeast cells that were transformed with each one of the viral proteins via the inducible pYZ1N plasmids [48] were plated on selected agar plates under the gene-repressing (gene-Off) or gene-inducing (gene-On) conditions (Fig. 3). Correct nucleotide sequence of each viral gene carried on the pYZ1N plasmid was confirmed by Sanger sequencing. *S. pombe* cells transformed with pYZ1N-Vpr or an empty pYZ1N plasmid were used as positive and negative controls, respectively. We have reported previously that Vpr prevents colony formation whereas pYZ1N plasmid has no cytotoxic effect on cells [34, 64]. As shown in Fig. 3, all HIV-1 viral gene-repressing (gene-Off) cells formed colonies as anticipated. On the gene-inducing (gene-On) plates, as expected, cells containing the empty pYZ1N plasmid formed colonies but HIV-1 Vpr prevented colony formation. Most of the HIV-1 viral gene expressions in fission yeast did not show any cytotoxic effects as they formed the same sizes of colony as the pYZ1N control. In contrast, however, the fission yeast strains expressing *rev* and *PR* showed much reduced or no colony formation indicating inhibition of cell growth or possible cytotoxic effects following expression of the respective genes.

### Effects of HIV-1 *rev* gene expression on yeast cell growth

**Fig. 4:**
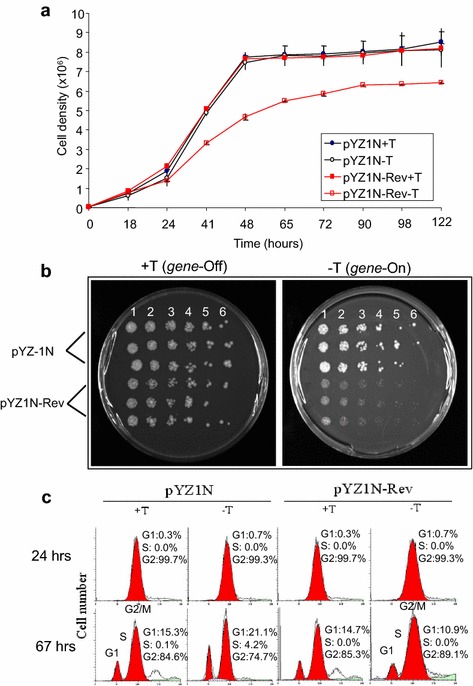
Effect of HIV-1 Rev protein production on fission yeast. **a** Expression of HIV-1 *rev* delays cellular growth of *S. pombe*. *S. pombe* cells containing pYZ1N-*rev* was grown under gene-repressing (+T) and gene-inducing (−T) conditions in liquid minimal selection EMM at 30°C. The cell density was measured over time and growth curves were plotted. *S. pombe* cell containing pYZ1N was used as a control. The experiment was repeated three times and the standard errors of each time point were calculated. **b** Rev delays colony formation in fission yeast cells. A semi-quantitative colony dot dilution assay was use to evaluate the ability of individual *rev*-expressing *S. pombe* cells to form colonies on agar plates. The pYZ1N-*rev* transformed *S. pombe* cells were grown in liquid EMM medium to log phase with thiamine. Thiamine was then removed from cells by washing and equal number of cells transferred to EMM supplemented plates with (gene-off, *left plate*) and without (gene-on, *right plate*) thiamine. The plates were incubated at 30°C for 5 days. Each colony on a plate from *left* to *right* (1–6) represents cells plated from approximately 1,000 to 3 cells following threefold dilutions. The pYZ1N plasmid was used as a control. **c** Rev does not affect cell cycle of fission yeast. Cell cycle profiles were measured in the pYZ1N-*rev* and pYZ1N control *S. pombe* cells by flow cytometric analysis. The data show analyses after 24 and 67 h of cell culture in EMM selective media with (gene-off) and without (gene-on) thiamine. The cell cycle stages were monitored after staining with propidium iodide. The *up-right numbers* are the percentage of cells in G1, S and G2 phases of the cell cycle.

To further differentiate the inhibitory effect of HIV-1 Rev protein on the cellular growth or toxicity of yeast cells, we measured the growth kinetics of *S. pombe* cells with and without *rev* gene expression. *S. pombe* cells containing pYZ1N-*rev* and pYZ1N control vectors were grown under gene-repressing (+T) and gene-inducing (−T) conditions in liquid minimal and selective EMM medium. Cellular growth was measured by the optical density (OD) over time from 0 to 122 h (5 days). At the first 24 h, both cells grown at the same pace with a doubling time of approx. 24 h. After 24 h of gene induction when the *rev* gene expression was fully expressed [34, 65], the growth velocity of Rev-producing cells became slower than those without *rev* gene expression that was grown actively in a logarithmic fashion (Fig. 4a). By 48 h, i.e., 2 days after *rev* gene induction, the difference in growth between *rev*-expressing and *rev*-repressing cells reached to the maximum level. As control, the pYZ1N transformed yeast cells showed no difference in growth of neither gene-repressing nor gene-inducing condition.

**Fig. 5:**
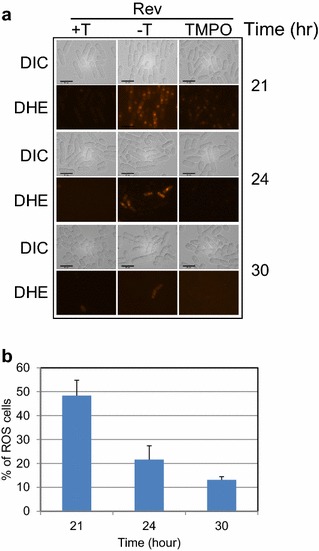
Expression of *rev* stimulates production of reactive oxygen species (ROS) in *S. pombe* cells. Fission yeast strain carrying pYZ1N-*rev* was grown in selective EMM either in the presence (+T) or absence (−T) of thiamine to suppressor or induce *rev* gene induction, respectively. **a** ROS production was detected by addition of a ROS indicator dye (DHE) to the culture medium right before microscopic observation overtime at different times as indicated. An oxygen radical scavenger (TMPO) was also added in separate test tubes but concurrently to test the specific production of ROS. TMPO was added twice during the course of the *rev* expression. TMPO was first added to the culture at the time of cell inoculation when *rev* gene expression was induced, and cells were treated again with TMPO when DHE was added to the culture to detect ROS production. *DHE* DHE-positive cells, *DIC* cells shown by differential interference contrast. *Scale bar* 10 μm. **b** Percentage of ROS-producing cells induced by HIV-1 Rev decreases over time. The % of ROS-producing cells induced by HIV-1 Rev was quantified by counting the numbers of DHE-positive cells over the entire cell population of three independent experiments.

### Rev causes growth delay but not cell death

Retarded cell growth could be an indication of cell death due to cytotoxicity. So we checked whether the growth delay caused by Rev was due to cell death. A semi-quantitative colony dot dilution assay was used, which quantified the ability of individual cells to form colonies on agar plate. All of the cells expressing *rev* formed colonies with expected numbers on each agar plate although the colony sizes were much smaller (Fig. 4b). This observation suggested that Rev causes growth delay but not cell death. Therefore, Rev causes growth delay but not cell death.

### Rev does not affect cell cycling of fission yeast

Because Rev affects cellular growth of fission yeast cells, we were interested in testing whether it affects the distribution of cell cycles. The cell cycle profile of *S. pombe* cells containing *rev*-expressing plasmid was monitored by flow cytometry. In standard EMM medium, *S. pombe* cells normally reside predominantly in the G2 phase of the cell cycle [66]. If Rev induces G1 delay, we should expect a shift of the predominantly G2 cell population to G1 after the production of Rev protein. Similarly, Rev-induced G2 delay should be represented by no shift of G2 cell population to G1 in the EMM medium. In this experiment, production of HIV-1 Rev protein did not change the cell cycle profile (Fig. 4c). Consistently, the pYZ1N vector was used as a control and showed no cell cycle difference between gene-repressing (+T) and gene-inducing (−T) conditions.

### Rev stimulates the production of reactive oxygen species (ROS)

To further explore the possible molecular mechanism underlying Rev-induced growth delay, possible intracellular stress induced by Rev was measured by probable production of ROS. A ROS-specific dye, DHE that produces red fluorescence in the presence of ROS, was used to measure possible cellular oxidative stress in *rev*-expressing cells. A ROS scavenger TMPO that can specifically remove free radicals by forming stable complexes [67], was also used as a control to verify the specific production of ROS. Expression of HIV-1 *rev* was induced in a fission yeast strain carrying the pYZ1N-*rev* plasmid, and the level of ROS was subsequently detected in cells at 21, 24, and 30 h after *rev* gene induction (Fig. 5). At 21 h, strong red fluorescence was detected in the *rev*-expressing cells; whereas little or no red fluorescence was observed in the *rev*-repressing cells (Fig. 5a). Consistently, treatment of the same *rev*-expressing cells with the ROS scavenger TMPO significantly reduced intensity of the red fluorescence suggesting the observed ROS production is Rev specific. Interestingly, Rev-induced ROS production faded away very quickly (Fig. 5b). About half of the *rev*-expressing cells produced the ROS signals 21 h after *rev* gene induction. Less than 30% of the cells showed ROS production 3 h later and a little more than 10% of those cells were DHE-positive by 30 h after gene induction. Together these data suggest expression of HIV-1 *rev* gene in fission yeast triggers intracellular oxidative stress. However, such a cellular stress response might be transient, which could potentially explain at least in part why those *rev*-expressing cells slowed cell growth but did not die.

### Effect of Rev protein on mammalian cell growth

**Fig. 6:**
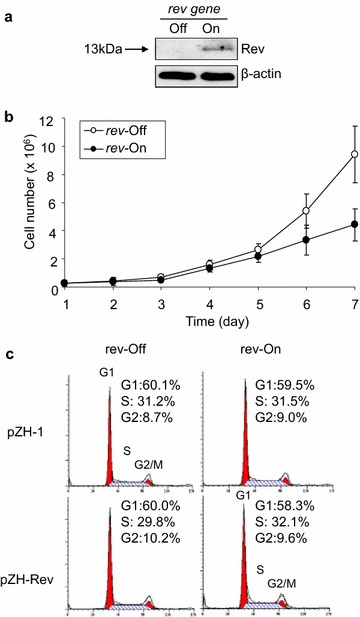
Effect of HIV-1 Rev in mammalian cells. **a** Stable expression of HIV-1 *rev* gene in 293VE632 cells. The inducible pZH-1-*rev* plasmid was transfected into 293VE632 cells. The *rev* gene was expressed following addition of muristerone A and confirmed by Western blot analysis using a HIV-1 Rev monoclonal antibody. Lane “Off”, *rev*-repressing 293VE632 cells; lane “On”, *rev*-expressing 293VE632 cells. Western blot shows a 13 kDa protein band that was reacted to the anti-Rev antibody. β-actin was used as a loading control. **b** The 293VE632 cells containing the pZH-1-*rev* plasmid were grown under gene-repressing (*rev*-Off) and gene-inducing (*rev*-On) conditions for up to 7 days. The cells were collected every 24 h, and the cell numbers were counted with Tryptan blue staining (Sigma-Aldrich) [69]. The growth curves were generated with an average of three different experiments. **c** Rev does not affect mammalian cell cycle. The 293VE632 cells transfected with pZH-1 and pZH-1-*rev* plasmids were cultured without muristerone A (*rev*-Off) or with muristerone A (*rev*-On). DNA contents of the cells were determined by flow cytometric analyses after 5 days of culture. The *upright numbers* are the percentage of cells in the G1, S and G2 phases of the cell cycle. The 293VE632 cells containing pZH-1 plasmid was used as a control (*upper row*).

Since HIV-1 Rev caused growth delay in *S. pombe*, we were interested in whether the same effect could also be observed in mammalian cells. In order to observe the effect of Rev protein on mammalian cellular growth, we created a mammalian gene inducible system to produce Rev. The HIV-1 *rev* gene was cloned into the mammalian cell expressing vector pZH-1 and transfected into 293VE632 cells [68]. The transfected cells were selected with hygromycin. After exerting drug selection for 2 weeks, Rev protein production was tested by inducing its expression with muristerone A. The cell lysate were collected and monoclonal anti-Rev antibody was used to confirm the expression of Rev protein (Fig. 6a). It was anticipated that if Rev affects mammalian cell growth, there should be a difference of cell numbers between *rev*-expressing and *rev*-repressing cells. The cell numbers of *rev*-containing 293VE632 cells were counted with Trypan blue staining under *rev*-repressing and *rev*-inducing conditions, and growth curves were generated. Indeed, there was a diverging growth differences between *rev*-expressing and *rev*-repressing cells after 5 days of culture (Fig. 6b). Finally, we tested whether the Rev-induced growth delay was due to, or at least partially due to alteration in cell cycling. To test this, 293VE632 cells containing pZH-1 and pZH-*rev* were cultured under the *rev*-repressing and *rev*-inducing conditions. Following gene induction for 5 days, the cells were harvested and the DNA contents were measured by flow cytometry as described in the materials and methods. No significant differences in cell cycle distributions were seen between *rev*-expressing and other control 293VE632 cells (Fig. 6c). Therefore, consistent with our observations in fission yeast, Rev also reduces mammalian cellular growth but does not affect its cell cycle distribution.

## Discussion

Maintaining faithful viral production during HIV-1 infection requires highly coordinated production of each individual viral protein and orchestration of the entire protein complexes. Each viral protein has to interact with host cellular proteins at defined subcellular sites to ensure successful completion each step of the viral life cycle. Identification of subcellular localization of all viral proteins encoded by HIV-1 is therefore a key step towards a comprehensive understanding of viral production and their potential effects on basic cellular functions. Here, we demonstrated the subcellular localizations of all HIV-1 viral proteins in fission yeast cells. This might be the first effort to illustrate the subcellular localizations of the entire HIV-1 genome all at once in a single cellular system. The use of GFP tagging allows the selective and specific detection of HIV-1 proteins at very low concentrations with good signal-to-background ratio. Combined information on HIV-1 viral protein subcellular localization with genome-wide protein–protein interaction data will be instrumental in establishing the interrelationships between the viral and host cellular proteins that determine virologic functions of the virus [70].

In fission yeast, we observed that HIV-1 P24, P7, P66, P51, IN, Gp120, Gp41, Vpr, Vif, Rev and Tat are all predominantly localized in the nuclei (Fig. 2). Please note that nuclear localization of Vpr has long been established in our laboratory [48]. It was used as a positive control here to confirm appropriate experimental conditions. The P17 and Vpu are primarily localized in the cytoplasm, which is consistent with previous findings in mammalian cells [59]. Also, the Gag precursor protein was seen to aggregate in the cytoplasm of unknown sites that resembles mammalian report [51]. The PR and Nef did not seem have any preference but distributed throughout the cell. Based on the previous reports on subcellular localizations of HIV-1 viral proteins in mammalian cells, there is a general correlation between fission yeast and mammalian cells (Table 1).

We further examined the potential growth or cytotoxic effects of the viral gene expressions on fission yeast cells. While most of the HIV-1 viral protein productions have no inhibitory effects on colony formation in fission yeast, Vpr, PR and Rev showed significant inhibitory effects (Fig. 3). The effect of Vpr has been studied extensively before [34, 37–40, 48]. Rev was chosen here for further analysis (Note: we have carried out subsequent and extensive studies on PR that will be described elsewhere).

HIV-1 Rev is an 18 kD phosphoprotein with 116 amino acids that contains a nuclear localization signal (NLS) and nuclear export signal (NES) [71, 72]. Thus it is capable of shutting between the cytoplasm and nucleus where it also specifically bind to the Rev responsive element (RRE) for nuclear transport of HIV-1 RNAs. In HIV-infected cells, HIV-1 Rev protein is located predominantly in the nucleus/nucleolus [73–75]. In addition, Rev is also a shutting protein between the nucleus and cytoplasm [75]. Thus it was not surprising that Rev was found to localize predominantly in the nucleus but also in the cytoplasm of the fission yeast cells (Fig. 2c, 5th row). Rev is a “nuclear regulatory protein” [76]. Mechanistically, Rev was found in the nucleus of mammalian and budding yeast cells where they both interact with a small nucleoporin-like protein hRIP/RAB1 and yRip1p, respectively [62, 77]. Thus it would be interesting in the future to test whether Rev also interact with a similar nucleoporin-like protein in fission yeast. In addition, we found that the production of HIV-1 Rev protein appeared to slow down cellular growth in both fission yeast and mammalian cells. However, Rev does not appear to induce cell death in proliferating cells but reduced the size of yeast colony formation (Fig. 4b) and the number of mammalian cells (Fig. 6b) presumably due to growth retardation. Consistently, there has been no report of Rev causing death in actively dividing mammalian cells. Interestingly, however, overproduction of Rev has been reported to cause death of non-dividing human cells [78]. In an attempt to resolve this potential discrepancy, we have explored possible molecular mechanism underlying Rev-induced growth delay revealed that expression of HIV-1 *rev* gene in fission yeast triggers transient production of ROS indicating fission yeast cells were experiencing intracellular oxidative stress upon Rev production (Fig. 5). Typically, upon induction of cellular oxidative stress, large amounts of ROS are released from mitochondria within the cell that often cause cell death [79, 80]. Paradoxically, no cell death was observed in the *rev*-producing cells. One possible explanation could be due to the rapid dissipation of the cellular stress response in continue dividing cells. Conceivably, possible accumulation of ROS production in non-dividing cells could potentially contribute to the cell death caused by Rev [78]. Test of this possibility will be our future goal of the study.

Even though Rev causes significant growth delay in fission yeast (Fig. 4a) and mammalian cells (Fig. 6b), it does not affect distributions of neither cell cycles (Figs. 3c, 6c). Altogether, our data suggested that Rev is a nuclear protein that reduces cellular proliferation in both fission yeast and mammalian cells. However, it does not affect cell cycling with minimal or no cytotoxic effect at least in proliferating cells. Future studies on molecular actions of HIV-1 Rev are important because expression of HIV-1 Rev protein is essential for completion of the viral life cycle [78, 81]. In particular, by localizing in the nucleus, Rev mediates nuclear export of partially spliced and un-spliced viral transcripts and its nuclear export signal allows nucleocytoplasmic shuttling [78, 81]. Therefore, Rev plays a pivotal role in viral replication. Conceivably, inhibition of its activities should block HIV-1 viral replication thus infection.

In summary, we successfully cloned and expressed all of the HIV-1 viral genes in *S. pombe.* By using a fission yeast inducible *nmt1* promoter, we were able to demonstrate the specific effects of viral proteins on fission yeast. In particular, we observed that, most of the viral proteins localized at the same or similar subcellular sites to human cells, suggesting fission yeast could be used as a simple and suitable model for future analysis of some of the HIV-1 proteins. Interestingly, besides HIV-1 Vpr, we found that HIV-1 PR and Rev also inhibit colony formation of fission yeast. Additional analysis of Rev suggested that there are a number of functional resemblances of the Rev effect between fission yeast and mammalian cells. These physical and similar effects on cellular functions suggest similar molecular mechanism of actions for HIV-1 Rev in yeast and mammalian cells, despite their evolutionary distance. Therefore, fission yeast might be suited as a model system for further investigation of this viral protein.

## Methods

### Cell and growth media

SP223, a wild-type *S. pombe* strain (*h*-, *ade6*-*216*, *leu1*-*32*, *ura4*-*294*), was used in this study [27, 34]. Standard YES complete and EMM minimal media supplemented with adenine, uracil, leucine or thiamine (20 µM) when necessary, were used for yeast cell culture and growth. Solid Luria–Bertani (LB) medium supplemented with Ampicillin (100 µg/ml) was used for competent *E. coli* Top 10 cell transformation. Mammalian 293VE632 cell was used to create a *rev*-inducible gene expression system. This is a stable zeocin-resistant mammalian cell line [68] expressing a heterodimer of the modified ecdysone receptor (VgEcR) and the retinoid X receptor. The heterodimer binds to a hybrid ecdysone response element (ECRE) only in the presence of the synthetic analogue of ecdysone, muristerone A, thus leading to induction of HIV-1 *rev* gene expression [82, 83]. The mammalian cell lines were maintained in Dulbecco’s modified Eagle (DMEM) medium containing 10% FBS, 100 µg/ml Zeocin (Invitrogen).

### Plasmids

pYZ1N and pYZ3N were used as previously described [48] for inducible viral gene expressions in fission yeast cells. These plasmids carry a *nomessage inthiamine* (*nmt1*) promoter. Under this inducible gene expression system, viral gene expression can be repressed or induced in the presence or absence of thiamine, respectively [65, 84]. These plasmids carry the *leu2* gene as a selection marker. In pYZ3N, GFP is fused to the viral gene at its 5′ end that was used to visualize intracellular location of each viral protein. For mammalian studies, a muristerone A-inducible gene expression pZH-1 plasmid was used as described previously [68].

### Molecular cloning of HIV-1 genes in fission yeast and mammalian cells

The plasmid pNL4-3, which carries an entire HIV-1 genome, was used as a template for viral gene cloning with the exception of *rev* and *tat* genes. The PSV-Rev plasmid, which carries a *rev* cDNA and originated from a HIV-1 cDNA clone pCV1, was used and obtained from the NIH AIDS Reagent Program [85]. The ptatC6H plasmid carrying the *tat* cDNA was used, which was a gift from Dr. David Pauza and originated from [86]. For mammalian studies, the *rev* gene was cloned in pZH-1 and gene expression was induced in 293VE632 cells by adding 1 µM muristerone A to the growth medium [68].

### Recombinant DNA transformation and gene expression

Each of the viral genes was PCR amplified, cloned and confirmed by Sanger DNA sequencing. The confirmed recombinant DNA was transformed into SP223 cells by electroporation using the BTX ECM600 protocol 0226 [87]. The transformants were selected on a minimal selective medium. For viral gene expression, a single yeast colony, which carries viral gene-containing plasmids, was grown to log phase on specific EMM liquid medium supplemented with 20 μM thiamine. The cells were then harvested and washed to remove the thiamine. Finally, 2 × 10^5^ cells/ml were grown and tested in fresh specific EMM liquid medium with thiamine (gene-Off) and without thiamine (gene-On). The cell suspensions were incubated at 30°C with constant shaking before observations.

### Subcellular localization of HIV-1 viral proteins in fission yeast

The HIV-1 viral proteins fused to GFP at their 5′ ends in pYZ3N were expressed and their subcellular localizations were visualized by fluorescent microscopy. Prior to visualization, the nucleus of the cells were stained with a nuclear dye 4′,6′ diamino-2-phenylindole (DAPI). Briefly, thiamine was washed out of a log phase culture with distilled water. The cells were inoculated in a thiamine free minimal media and incubated for at least 14 h for gene expression. The cells were then collected by spinning at 3,000 rpm for 5 min and re-suspended in suitable EMM medium. For DAPI staining, 5 µl of the cell suspension was pipetted onto a glass slide. The cells were heat fixed for 1 min at 70°C on a hot plate. The slide was then cooled down for a few seconds before counterstained with 1 µg/ml final concentration of DAPI. A coverslip was applied and the cells visualized under a fluorescence microscopy. A Leica DMR fluorescence microscope equipped with a high performance charge-coupled device camera (Hamamatsu) and Open-Lab software (Improvision, Inc., Lexington, MA, USA) was used for all imaging analysis. For DAPI, the cells were observed with a Leica A8 filter with an excitation of 360 nm (ranged from 340 to 380 nm) and emission of 470 nm (range, 450 to 490 nm). For the observation of GFP, a Leica L5 filter, which has an excitation of 480 nm (ranged from 460 to 500 nm) and emission of 527 nm (ranged from 512 to 542 nm), was used.

### Effect of HIV-1 viral proteins on yeast colony formation

A single yeast colony, which carries viral gene-containing plasmids, was picked from a selective minimal plate and inoculated overnight in specific EMM liquid medium supplemented with thiamine. The following day, 1 ml of a mid-log phase culture was spin down, washed three times with distilled water and the cells re-suspended in appropriate volume of water. About 1–5 µl were streaked onto selective EMM plates with and without thiamine. The plates were incubated at 30°C for 6 days to observe for colony formation and numbers of cells within each colony (at low dilutions) as a semi-quantitative indicator of the viral effect on cellular growth or cytotoxicity.

### Characterization of HIV-1 Rev protein activities in fission yeast

Apart from the colony formation assay on agar plates, the effect of Rev on yeast cell proliferation was also determined by growth kinetics analyses as previously described [34]. Cell cycle profile of *r*ev-expressing cells was measured by flow cytometry using a previously described method [31, 49]. A semi-quantitative growth and colony dot test was also used to quantify the effect of Rev on yeast cellular growth and colony formation. Briefly, the cells were grown to a log phase in liquid EMM as previously described and washed to remove thiamine. Cell suspension of 5 × 10^5^ cells/ml was made on a 96 well plate and five consecutive threefold dilutions were made. Using a multiple pipette, 2 µL spots were generated on EMM supplemented media with (gene-off) and without (gene-on) thiamine. The plates were incubated at 30°C for 5 days to observe for colony formation. Fission yeast cells that were transformed with an empty pYZ1N vector were used as control. Potential effect of Rev on cellular oxidative stress was measured by production of reactive oxygen species (ROS), which can be detected by a ROS-specific dye, dihydroethidium (DHE) that produces red fluorescence in the presence of ROS as described previously [39]. A ROS scavenger, 3,3,5,5-tetramethylpyrroline N-oxyde (TMPO) was used as a control to specifically remove ROS [71].

### Characterization of Rev-specific activities in mammalian cells

A Rev protein-inducible mammalian system was created and used for growth kinetics measurement as well as for analysis of cell cycle profile. For growth kinetics measurement, the Rev protein was expressed in transfected 293VE632 cells following the addition of muristerone A. Protein expression was confirmed by Western Blot analysis using a monoclonal anti-Rev antibody as previously described [40]. Viable cells were grown in equal numbers in both *rev*-inducing and *rev*-repressing conditions for 7 days. The number of viable cells were counted by observation of differential stainings of the Trypan Blue (Sigma-Aldrich) [69]. Finally, for cell cycle profile measurement, 293VE632 cells containing pZH-1 (control cells) and pZH-1-*rev* were cultured under *rev*-repressing and *rev*-inducing conditions as mentioned before. Following gene induction of 5 days, the cells were harvested and prepared for flow cytometry as described [40, 41]. The DNA contents of the cells were analyzed on a FACScan flow cytometry device (Becton–Dickinson) using the Cell Quest software.
